# The participation rate of migrant women in gestational diabetes screening in Austria: a retrospective analysis of 3293 births

**DOI:** 10.1007/s00404-018-4964-5

**Published:** 2018-11-21

**Authors:** Christoph Weiss, Peter Oppelt, Richard Bernhard Mayer

**Affiliations:** 1 Department of Gynecology, Obstetrics and Gynecological Endocrinology, Kepler University Hospital, Johannes Kepler University Linz, Altenberger Strasse 69, 4040 Linz, Austria; 2 Kepler Universitätsklinikum, Krankenhausstrasse 26–30, 4021 Linz, Austria

**Keywords:** Gestational diabetes mellitus, Migrants, Oral glucose tolerance test, Screening, Participation rate, Prevalence

## Abstract

**Purpose:**

This study evaluated the extent to which migrant women participate in the mandatory oral glucose tolerance test (OGTT) for gestational diabetes mellitus (GDM) screening in Austria.

**Methods:**

A retrospective data analysis was carried out of births at an obstetrics unit in a university hospital between January 2013 and December 2015. The inclusion criteria were singleton pregnancies, live births, birth weight ≥ 3500, and no preexisting diabetes mellitus. The patient’s extramurally obtained OGTT values and history of GDM were checked. If the mother’s country of birth was not Austria, the woman was classified as a migrant. Three groups were defined: group 1—women with normal OGTT; group 2—women with pathological OGTT; and group 3—women without OGTT or with an incomplete OGTT.

**Main outcome measures:** Numbers of complete and incomplete OGTTs and rate of women with pathological OGTTs not treated in accordance with the guidelines among mothers born in Austria or migrants. The groups were compared using the *t*-test, chi-squared test, or Fisher’s exact test.

**Results:**

A total of 3293 births met the inclusion criteria, and 43.52% of all mothers were migrants; 16.8% of all women had pathological OGTT findings. Only 60.1% of the latter received treatment in accordance with the guidelines. The proportion of mothers born in Austria who did not have OGTTs, or only incomplete ones, was 5.4%. In the group of migrant women, the corresponding figure was 10.5% (*P* < 0.01).

**Conclusions:**

Migrant women have significantly lower rates of participation in GDM screening.

## Introduction

In recent years, war, terrorism, and natural disasters have led to major waves of migration to Europe. The large numbers of migrant women in need of care, most of them at reproductive age, represent an increasing challenge for the health-care systems in the countries affected [1].

On the one hand, the refugees need to be provided with the best possible medical care, and the aim is to integrate them into existing screening programs. On the other hand, treating migrant women is difficult in everyday practice due to language barriers and the different beliefs they have about health and illness [2]. This applies in particular to pregnant migrants. Many of these women have never experienced any comparable preventive medical examinations in their home countries [3].

It has long been known that there is an association between impaired glucose tolerance during pregnancy and increased maternal and fetal morbidity and mortality rates. Impaired glucose tolerance leads to more macrosomic fetuses, resulting in increased rates of cesarean deliveries, shoulder dystocia, and birth injuries. Neonates of mothers with impaired glucose tolerance experience more clinical neonatal hypoglycemia and more hyperbilirubinemia and hypocalcemia. In addition, both the affected women and their children have an increased risk of developing manifest diabetes mellitus [4–6]. The affected pregnancies are considered to be high-risk, and the women require close monitoring, early treatment, and monitoring on a long-term basis after delivery.

Gestational diabetes mellitus (GDM) is defined as a glucose tolerance disorder that is first recognized during pregnancy. According to the literature, an estimated 10% of all pregnant women in Austria are affected [7]. In one national prospective multicenter study including 1466 women, a prevalence of 21% was found in a risk group [8]. Accurate data on the prevalence are not available for Austria, and the same also applies to most other European countries [9]. Overall, the prevalence of GDM is increasing internationally, and it represents a growing problem for national health-care systems not only because of the associated long-term sequelae of the condition [10, 11]. It has been shown that treatment for GDM is capable of significantly reducing fetal and maternal complications. Treatment may include dietary measures, physical activity, or insulin therapy [12, 13].

Numerous studies have shown that migrant women are at high risk for developing GDM [14]. The rate of impaired glucose tolerance in pregnancy is significantly higher in some migrant populations, leading to increased perinatal complications in comparison with mothers without a migrant background. This applies, in particular, to women from South-East Asia and North Africa [15]. In addition, migrants with GDM are at greater risk for developing diabetes mellitus in later life in comparison with nonmigrants with GDM [16].

The oral glucose tolerance test (OGTT) has long been a gold standard in the diagnosis of GDM. In recent decades, however, many different test methods and cut-off values for the definition of GDM have been published and included in numerous recommendations and guidelines [17]. Evidence-based thresholds for predicting neonatal outcomes were only established in 2008, based on the Hyperglycemia and Adverse Pregnancy Outcome (HAPO) study including more than 25,000 pregnancies [4, 18].

Today, the World Health Organization and most medical societies define GDM using values greater than or equal to a fasting plasma glucose level of 92 mg/dL, plasma glucose of 180 mg/dL 1 h or 153mg/dL 2 h after a 75-g oral glucose load [17]. One pathological value is sufficient for a diagnosis of GDM. Diabetes mellitus is considered when the fasting glucose value is ≥126 mg/dL or a random glucose value ≥ 200 mg/dL is measured. On the basis of these findings, mandatory GDM screening was introduced for the Austrian maternity registry in 2010. Testing is to be carried out between 25 + 0 and 28 + 0 weeks of gestation [19].

This study investigated the extent to which women with a migrant background participate in screening for GDM in pregnancy screening programs in Austria. To the best of our knowledge, there have not as yet been any reports on this issue. Perinatal outcomes and modes of birth were also compared between women with and without OGTT. Birth weight, umbilical artery pH (apH), the 5-min Apgar score and admission to a neonatal care unit were used as fetal outcome parameters.

## Materials and methods

A retrospective data analysis including all births between 1 January 2013 and 31 December 2015 was carried out. All of the children were born in the Department of Gynecology, Obstetrics and Gynecological Endocrinology at Kepler University Hospital, Linz, Austria. A total of 10,911 children were delivered during the study period, with a mean birth weight of 3245 g (SD 656 g). The inclusion criteria were: single pregnancy, live birth, birth weight ≥ 3500 g with complete biometric records for the fetuses and mothers, and no preexisting diabetes mellitus.

To make manual data collection of OGTT values feasible, the number of births to be included in the study had to be restricted sensibly. A birth weight of ≥3500 g was selected as a reasonable cut-off value. This is because fetal macrosomia is a major risk for undetected or untreated GDM [20]***.*** Fetuses with a birth weight ≥ 4500 g, in particular, are associated with an increased risk for perinatal mortality and morbidities such as shoulder dystocia, brachial plexus injuries, fractures, and asphyxia [21]. Selecting 3500 g as a lower limit ensured that all such fetuses at risk would be included. It could be argued that excluding fetuses with a birth weight  < 3500 g might lead to underrepresentation of high-risk populations for GDM, e.g., mothers from South-East Asia who naturally give birth to lighter babies. An analysis of 21,677 deliveries in our department from 2011 to 2016 showed that only 123 mothers (0.5%) were born in South-East Asia. In the study group, only 12 women (0.4%) were born in the region (*P* = 0.14). This subgroup is thus of very little relevance in the population as a whole.

Perinatal data for the neonates (birth weight, apH, 5-min Apgar score, transfer to a neonatal care unit, and mode of delivery) were taken from the in-house database. The data were entered by the midwives after the births.

Maternal data (height, weight before pregnancy and country of birth) and the OGTT values were taken from the maternity records. These entries were made by physicians in private practice during the mandatory maternal examinations. All OGTTs were performed and interpreted extramurally. As part of our outpatient contacts, all available OGTT values are routinely transferred to the patient record card. For data analysis, these entries had to be read out manually from the filed paper records.

If the mother’s country of birth was not Austria, the woman was classified as a migrant. Information about how long the women had already been living in Austria was not collected.

In the context of outpatient contacts, women are routinely asked about blood sugar controls and further check-ups in case of pathological OGTT values. In accordance with the applicable guidelines in Austria, women with a pathological OGTT must receive information about GDM, its possible sequelae for mother and child, and the therapeutic options available (lifestyle modification, medication) in a detailed information discussion. In addition, nutritional counseling and initial self-monitoring of blood glucose during the first 2 weeks of care should be mandatory [22].

Pathological OGTT results that had not been checked were noted in the patient record card. These entries were also read out manually for this study.

The following classification was chosen for the outcome analysis:

- Group 1: women without impaired glucose tolerance (normal OGTT).
- Group 2: women with impaired glucose tolerance (pathological OGTT).
- Group 3: women without an OGTT or with an incomplete OGTT.

Women with incomplete testing were assigned to group 3 only if none of the available OGTT values were pathologically elevated.

### Statistical analysis

Statistical analysis was carried out using the R statistical software package [23]. Normally distributed data and comparisons between the groups were analyzed using a two-sided *t* test. The Chi-squared test or Fisher’s exact test were used for nominal data. Statistical significance was set at *P* < 0.05 for all the analyzes.

## Results

In all, 3293 births met the inclusion criteria. In the overall study group, 56.48% of all mothers were born in Austria. Thus, 43.52% were migrants, and 31.7% of these women came from outside the European Union. The 15 most common countries of origin are listed in Table 1.

**Table 1:** The 15 most common countries of origin of migrant women and corresponding proportions of impaired glucose tolerance and no oral glucose tolerance testing (OGTT) or incomplete testing

Country of birth	*n*	%	Impaired glucose tolerance (%)	No OGTT or incomplete OGTT (%)
Austria	1860	56.48	15.81	5.43
Migrants (total)	1433	43.52	18.21	10.47
Bosnia	248	7.53	14.92	6.45
Turkey	182	5.53	28.57	8.24
Romania	168	5.10	19.64	12.50
Kosovo	131	3.98	12.21	14.50
Germany	72	2.19	13.89	8.33
Macedonia	51	1.55	19.61	15.69
Chechnya	48	1.46	16.67	20.83
Serbia	41	1.25	17.07	24.39
Hungary	40	1.21	20.00	5.00
Afghanistan	34	1.03	14.71	2.94
Croatia	31	0.94	9.68	19.35
Former Yugoslavia	27	0.82	14.81	0.00
Russia	27	0.82	7.41	11.11
Poland	22	0.67	13.64	9.09

The migrants were significantly younger and already had more children than women born in Austria. There were no differences with regard to body mass index (BMI). The maternal characteristics are summarized in Table 2.

**Table 2:** Characteristics of the overall study group

	Women born in Austria (*n* = 1860)	*P*	Migrants (*n* = 1433)
Age (years; mean ± SD)	30.4 ± 5.3	< 0.01	29.4 ± 5.11
BMI (kg/m^2^; mean ± SD)	24.7 ± 5.4	0.28	24.5 ± 4.88
Parity		< 0.01	
1	805 (43.28%)		482 (33.64%)
2	806 (43.33%)		523 (36.50%)
3	187 (10.05%)		261 (18.21%)
4	38 (2.04%)		100 (6.98%)
5	16 (0.86%)		41 (2.86%)
≥ 6	8 (0.43%)		7 (1.81%)
Fetal birth weight (g; mean ±SD)	3818 ± 251,6	< 0.01	3843 ± 274,51
Duration of pregnancy (days; mean ±SD)	281.41 ± 7.19	0.41	281.20 ± 7.17

*BMI* body mass index, *SD* standard deviation

### Groups

*Group 1* (*n* = 2487): Fully conducted glucose tolerance testing with normal results was carried out in 75.1% of the women included, 58.9% of whom were born in Austria.

*Group 2* (*n* = 555): GDM was diagnosed and treated in accordance with the current guidelines in 10.1% of all the women included, 74.0% of whom received management with lifestyle modifications, while 26.0% needed insulin therapy.

A further 6.7% of all the women included had pathological OGTTs but did not receive any further medical care. A total of 16.8% of the mothers thus met the definition of GDM. Pathological OGTT results for which follow-up care had not been provided were noted in 7.1% of Austrian women and 6.1% of migrants. Maternal and fetal characteristics of women with and without treatment in accordance with the guidelines are summarized in Table 3.

**Table 3:** Maternal and fetal characteristics of the women (*n* = 221) who had pathological OGTTs but did not receive any further medical care as recommended by the guidelines and those women (*n* = 334) in which GDM was treated in accordance with the guidelines

	Women without treatment		Women with treatment
Age (years; mean ± SD)	30.67 ± 5.41	ns	30.88 ± 5.44
Parity (mean ± SD)	1.92 ± 1.06	*p* = 0.04	2.13 ± 1.29
BMI (kg/m^2^; mean ± SD)	26.07 ± 5.47	*p* < 0.01	27.96 ± 6.39
Women born in Austria	133 (60.38%)	*p* < 0.01	161 (48.20%)
Migrant women	88 (39.62%)	*p* < 0.01	173 (51,80%)
Fetal birth weight (g; mean ± SD)	3846 ± 275.91	ns	3881 ± 292.89
Cesarean section rate (%)	30.76%	ns	31.14%
apH (mean ± SD)	7.25 ± 0.07	ns	7.25 ± 0.08
5-min Apgar score (mean)	9.71	ns	9.68
Transfer to neonatal care unit (%)	13.57	*p* = 0.03	20.96

*apH* umbilical artery pH, *BMI* body mass index, *OGTT* oral glucose tolerance test, *SD* standard deviation

Impaired glucose tolerance was present in 15.8% of the Austrian mothers and 18.2% of migrant women (*P* = 0.01). There were considerable differences in the prevalence of impaired glucose tolerance among migrant women relative to the country of origin (Table 1).

*Group 3* (*n* = 251): In the study group, 7.6% of the mothers did not receive an OGTT or did not have a complete OGTT; 59.8% of these women were migrants, and 39.8% came from outside the European Union. The proportion of mothers born in Austria who did not have OGTTs or only incomplete testing was 5.4%. In the group of migrant women, the corresponding figure was 10.5% (*P* < 0.01) (Table 1).

There were significant differences (*P = *0.01) in age and body mass index (BMI) between groups 1 and 2. There were no differences between groups 1 and 3. The same applies to parity. There was a significant difference (*P* < 0.01) in the proportions of migrants in the groups; group 3 contains the most migrants, at 59.76%. Women with impaired glucose tolerance (group 2) had a higher risk of delivering by cesarean section than women with normal OGTTs (group 1) (31.0% vs. 18.3%, *P* < 0.01). There were no significant differences between groups 1 and 3 (*P* = 0.81) (Table 4).

**Table 4:** Maternal and fetal characteristics of the three different groups

	Group 1 (normal OGTT) *n* = 2487	Group 2 (pathological OGTT) *n* = 555	Group 3 (no OGTT or incomplete) *n* = 251
Age (years; mean ± SD)	29.86 ± 5.13	30.80 ± 5.43	29.46 ± 5.47
Parity	1.87 ± 0.95	2.05 ± 1.21	2.23 ± 1.24
BMI (kg/m^2^; mean ± SD)	24.15 ± 4.77	27.21 ± 6.12	24.06 ± 4.90
Women born in Austria	1465 (58.90%)	294 (52.97%)	101 (40.24%)
Migrant women	1022 (41.10%)	261 (47.03%)	150 (59.76%)
Fetal birth weight (g; mean ± SD)	3823 ± 259	3867 ± 287	3803 ± 227
Cesarean section rate (%)	18.29	30.99	19.12
apH (mean ± SD)	7.25 ± 0.07	7.25 ± 0.07	7.25 ± 0.07
5-min Apgar score (mean)	9.80	9.68	9.74
Transfer to neonatal care unit (%)	13.72	21.98	14.61

*apH* umbilical artery pH, *BMI* body mass index, *OGTT* oral glucose tolerance test, *SD* standard deviation

### Fetal outcome

The mean birth weight was 3829 g. Children of migrant women were significantly heavier than children of women born in Austria (Table 2).

Women with impaired glucose tolerance (group 2) had significantly higher fetal birth weights than those without impaired glucose tolerance (group 1; *P* < 0.05). There were no significant differences in fetal birth weight in group 3 in comparison with group 1 (*P* = 0.20) (Table 4).

There were no significant differences between the three groups with regard to apH (Table 4).

A significant difference in 5-min Apgar scores (*P* < 0.05) was noted between mothers with normal OGTTs (group 1) and those with abnormal OGTTs (group 2). There were no differences between groups 1 and 3 (*P* = 0.21) (Table 4).

There was a significant difference (*P* < 0.05) between group 1 and group 2 with regard to the rate of transfer of neonates to a neonatal care unit. There were no differences between groups 1 and 3 (*P* = 0.83).

Table 4 summarizes the maternal and fetal characteristics of the three groups.

## Discussion

It is well known from numerous studies that ethnicity has a significant impact on the prevalence of GDM [14], and this is also taken into account in the relevant guidelines [24]. The special needs of migrant women are increasingly coming into the focus of scientific research [25], and the corresponding findings are increasingly being implemented in clinical practice.

Despite the numerous studies published on the relationship between migration and GDM and its sequelae and risks for mother and child, there are no reliable data in the literature on the extent to which migrant women take part in the screening programs provided. In addition, there are no valid data on the prevalence of GDM in Austria.

The present study for the first time investigated the rate of participation by migrant women in the obligatory GDM screening program in Austria. At 7.6%, the overall rate of women who did not undergo an OGTT or did not have complete oral glucose testing was astonishingly high. As expected, migrants were significantly overrepresented. The proportion of mothers born in Austria who did not have OGTTs or only incomplete testing was 5.4%. In the group of migrant women, the corresponding figure was 10.5%. It can be assumed that providing information materials in the women’s native languages could improve participation rates. Surprisingly, there were no significant differences in maternal and fetal outcome parameters in comparison with women with normal OGTTs. There were also no differences between groups 1 and 3 with regard to the rate of cesarean sections. In our view, this result is best explained by the low numbers of cases in group 3. Assuming a similar prevalence of approximately 17%, only 42 women with undetected GDM would fall into group 3. The figure might be even lower, considering the fact that women with two normal OGTT values were also included in this group.

Overall, 10.1% of the women included in this study had a diagnosis of GDM and had been treated in accordance with the current guidelines. This rate shows very good consistency with the estimates for Austria cited in the literature. Despite pathological OGTT values, however, 6.7% of the women did not receive further guideline-based medical care. Interestingly, women born in Austria were more affected by this than migrants, at 7.1% and 6.1%, respectively. In comparison with women who received GDM treatment in accordance with the guidelines, fetuses of women without such care showed no significant differences with respect to fetal birth weight, cesarean section rates or umbilical cord arterial pH (Table 3). In our opinion, the generally very moderate elevated or only marginal OGTT-values in women without further care are responsible for not showing significant differences at such a sample size.

A total prevalence of impaired glucose tolerance as high as 16.8% was thus found in the study group. This is significantly higher than the general estimates given in the literature. One reason for the surprisingly high rate of almost 7% of women with pathological OGTT results who did not receive follow-up care could be the fact that different definitions of GDM have been published in recent years. Providing targeted information for specialists and for family physicians in particular could certainly improve the situation. Unfortunately, a lack of adequate postpartum follow-up for women with GDM is also observed internationally as well [26]. An analysis from an Austrian laboratory found that postpartum OGTT follow-up screening had been performed in only 4.2% of all women with GDM [27]. No data are available on the extent to which migrant women take part in postpartum OGTT screening. On the basis of the present data, it can be assumed that women with a migrant background are overrepresented in that area as well. This is particularly worrying, as migrants have an increased risk of developing diabetes mellitus after GDM [28]. Improving this situation should be a goal for all health-care professionals involved in pregnancy care. In addition, family physicians must increasingly be included in follow-up care for women with GDM.

The women included in this study were drawn from the risk group generally present in our department. This becomes clear from comparison with data from the Austrian birth registry. During the study period, the median birth weight in Austria was 3345 g, with an average cesarean section rate of 31.0%. The mean rate of transfers to a neonatal care unit was only 6.8% [29]. During the same period, the median birth weight in our department was 3330 g, with a mean cesarean section rate of 28.8%, and 21.8% of all newborns were transferred to the neonatal care unit. Nevertheless, the analysis confirms known data for maternal and fetal parameters. Thus, the findings also showed that the GDM risk for Turkish migrants was almost twice as high as that of women born in Austria [16, 30].

The retrospective study design must certainly be regarded as a limiting factor in the present study. In addition, women who had only recently arrived in Austria, and therefore, often lacked full maternity records could not be included in the study. Pregnant refugees are thus certainly underrepresented in the group. It was also not possible to establish clear distinctions between different ethnic groups, e.g., women born in Austria with a Turkish family background. The length of time for which the individual women had already been living in Austria was also not recorded.

Despite the increased risk of GDM and its consequences, migrant women often do not undergo GDM screening. In everyday practice, this should lead to an increased focus on this group of women and the provision of more targeted information about GDM and its sequelae. Furthermore, health-care professionals should offer OGTT more actively to migrant women to improve their participation rate in this important screening procedure. As a secondary finding in this study, it was noted that the prevalence of GDM in Austria is likely to be much higher than the estimates reported in the literature. For the future, a national, unselected survey of pathological OGTT values ​​would be preferable to allow better estimates of GDM. Surveying diagnosed GDM cases alone is not sufficient, due to the large number of unreported cases and women who do not receive follow-up care.
